# CDK7 is a prognostic biomarker for non-small cell lung cancer

**DOI:** 10.3389/fonc.2022.927140

**Published:** 2022-09-23

**Authors:** Christiane Kuempers, Tobias Jagomast, Carsten Heidel, Finn-Ole Paulsen, Sabine Bohnet, Stefanie Schierholz, Eva Dreyer, Jutta Kirfel, Sven Perner

**Affiliations:** ^1^ Institute of Pathology, University Hospital Schleswig-Holstein, Luebeck, Germany; ^2^ Department of Surgery, Schoen Klinik Neustadt, Holstein, Germany; ^3^ Department of Oncology, Hematology and Bone Marrow Transplantation with Division of Pneumology, University Medical Center Hamburg-Eppendorf, Hamburg, Germany; ^4^ Department of Pulmonology, University Hospital Schleswig-Holstein, Luebeck, Germany; ^5^ Department of Surgery, Medical University of Schleswig-Holstein, Luebeck, Germany; ^6^ Pathology, Research Center Borstel-Leibniz Lung Center, Borstel, Germany; ^7^ German Center for Lung Research (DZL) Department Borstel, Borstel, Germany

**Keywords:** CDK7, non-small cell lung cancer (NSCLC), prognostic, biomarker, immunohistochemistry

## Abstract

**Aim:**

Non-small cell lung cancer (NSCLC) remains the leading cause of cancer-related death globally despite promising progress of personalized therapy approaches. Cyclin-dependent kinase 7 (CDK7) is a kinase involved in transcription, overexpressed in a broad spectrum of cancer types and found to be associated with an unfavourable prognosis. In this study, we aimed to investigate the protein expression of CDK7 in a large cohort of NSCLC incorporating adenocarcinomas (adNSCLC) and squamous cell carcinomas (sqNSCLC) and to correlate its expression with clinicopathological data.

**Methods:**

We performed immunohistochemical staining of CDK7 on our cohort of NSCLC including 258 adNSCLC and 101 sqNSCLC and measured protein expression *via* a semi-automated read out. According to the median value of CDK7 the cohort was stratified in a CDK7 high and low expressing group, respectively, and results were correlated with clinico-pathological data.

**Results:**

CDK7 was significantly higher expressed in sqNSCLC than in adNSCLC. In the group of sqNSCLC, CDK7 expression was significantly higher in sqNSCLC with lymph node metastases than in sqNSCLC with N0 stage. We found a significantly worse overall survival and disease-free survival for patients with CDK7 high expressing NSCLC.

**Conclusion:**

Since a high CDK7 expression seems to be linked with a poor prognosis it might serve as a promising novel prognostic biomarker and its assessment could be implied in future routine diagnostic workup of NSCLC samples. Considering that CDK7 inhibitors are currently tested in several trials for advanced solid malignancies, it may also be a new target for future anti-cancer therapy.

## Introduction

Non-small cell lung cancer (NSCLC) remains, despite promising progress of personalized therapy approaches, the leading cause of cancer-related death globally (1).

The cyclin-dependent kinases (CDK) are a family of several serine/threonine kinases that regulate essential cellular processes. They are broadly divided into the two major

Subclasses of cell cycle–associated CDKs and transcription-associated CDKs, the latter group including CDK7. The transcription-associated CDKs play major roles in the multistep process of RNA polymerase II transcription. CDK7 is a 346 amino acid protein that binds to cyclin H and the accessory protein MAT1 to function as a CDK-activating kinase. CDK7 regulates the initiation of transcription and promoter escape by phosphorylating the carboxy-terminal domain of RNA Pol II and therefore has a general role in transcription (2). Overexpression of CDK7 is commonly observed in a broad spectrum of human cancers for example breast cancer, hepatocellular carcinoma, gastric cancer, colorectal cancer, squamous cell carcinomas of the oral cavity, head and neck (HNSCC) and esophagus, as well as ovarian cancer (3–10). Here, it was found to associate with aggressive clinicopathological features and unfavourable prognosis, except for ER+ breast cancer where its overexpression is linked with a better prognosis (11).

Due to CDKs control processes critical for cancer cell survival and growth, they have been discussed as promising therapeutic targets. Elevated CDK7 expression in tumor cells compared with their normal counterparts raise the possibility that tumors with increased expression of CDK7 may be more sensitive to CDK7 inhibition, particularly in the case of ER+ breast cancer, where the CDK7-activated nuclear receptor, ERα, drives tumor progression (12). However, there does not seem to be clear evidence in the literature regarding the question of whether protein expression of CDK7 is related to sensitivity towards CDK7 inhibitors.

Until now, even multiple CDK inhibitors (CDKIs) have been developed and tested in different cancer types (2, 12). CDKIs, specifically the ones that block the enzyme activity of CDK4 and CDK6, have been approved by FDA for the treatment of metastatic hormone receptor-positive breast cancer (13).

Concerning lung cancer, THZ1, a selective CDK7 covalent inhibitor, has recently been shown to be effective in reducing the expression of superenhancer-associated genes and inhibiting growth of small cell lung cancer (SCLC) (14). The more specific CDK7 inhibitor YKL-5–124 was found to predominately disrupt cell cycle progression while simultaneously triggering immune response signaling in SCLC, which provides a rationale for new combination regimens consisting of CDK7 inhibitors and immunotherapy (15).

There are also some studies on CDK7 inhibition for NSCLC (1, 16–18). Cheng et al. (16) could show that treatment with THZ1 suppressed proliferation and migration of human NSCLC cell lines, arrested cell cycle at G2/M phase and induced apoptosis. They found that CDK7 inhibition blocked the glycolysis pathway without affecting glutamine metabolism, suggesting that the inhibitory effect of THZ1 is due in part to an impairment of cancer metabolism. Hur et al. (18) investigated the effects of THZ1 in squamous cell carcinoma cell lines with SOX2 amplification and also found that THZ1 treatment led to suppression of cell growth and apoptotic cell death. They conclude that THZ1 may effectively control the proliferation and survival of SOX2-amplified squamous cell carcinoma cells through a decrease in global transcriptional activity. Ji et al. (17) found that THZ1-tolerant cells partially recovered their sensitivity to 3rd generation EGFR-TKIs and conclude that CDK7 inhibitors could potentially be used as a therapeutic strategy to overcome EMT-associated EGFR-TKI resistance in NSCLC.

However, the CDK7-mediated mechanisms involved in progression of NSCLC are not fully understood and protein expression of CDK7 in NSCLC has not extensively been studied so far.

In this study, we aimed to investigate the protein expression of CDK7 in a huge cohort of NSCLC incorporating pulmonary adenocarcinomas (adNSCLC) and pulmonary squamous cell carcinomas (sqNSCLC) and to correlate its expression with clinicopathological data and survival.

## Material and methods

### Cohort

359 patients with lung cancer (258 adNSCLC, 101 sqNSCLC) undergoing surgical resection were enrolled in this study. The median age of the patients (157 female, 202 male) at initial diagnosis was 67 years. At the time of the last follow-up, 56 patients were alive and 294 were deceased. Tumors were graded according to the 2015 World Health Organization Classification of Lung Tumors. 6 of the primary tumors were graded as G1 (1.7%), 176 as G2 (49%), and 175 as G3 (48.7%). In 3 cases (0.8%) grading was not provided. For determination of tumor state, the 8th Edition of UICC/TNM staging system was used. From primary tumors, 160 (44.6%), 95 (26.5%), 59 (16.4%) and 43 (12%) were classified as pT1, pT2, pT3 and pT4, respectively. In 2 cases (0.6%) note concerning T-stage was missing. In 31 cases (23 adNSCLC and 8 sqNSCLC, respectively) note concerning N-stage was missing. Archived tissue blocks and slides were collected from 2005 to 2017. All data were anonymized before inclusion in this retrospective study cohort.

This study was approved by the Internal Review Board of the University of Luebeck (file number 16-277, 16-278).

### Immunohistochemistry (IHC)

IHC staining was performed according to the manufacturer’s instructions, using the Ventana Discovery (Ventana Medical System) automated staining system. In brief, slides were incubated with a primary CDK7- antibody (mouse monoclonal, CDK7 (MO1) Mouse mAb #2916, 1:100, Cell Signaling, Danvers, MA, USA).

For IHC, tissue microarrays (TMA) were constructed from formalin-fixed paraffin-embedded (FFPE) tumor blocks as described previously (19). In short, for TMA construction each sample was represented in triplicates of 0.6 mm diameter cores. A tumor sample was incorporated in further analysis if at least one core was evaluable. Staining was considered positive if staining was nuclear.

Stained slides were scanned (Panoramic Desk, 3DHistech) and evaluation of the staining intensity was performed with the bioimage analysis software *QuPath*, short for Quantitative Pathology (20, 21). This software allows objective assessment of the staining intensity in different cellular compartments within specified regions of interest (ROI). ROIs were defined as tumor cell areas that were annotated in each TMA core to exclude stromal cells and benign areas from evaluation (**Supplement Figure 1**). Due to nuclear staining of CDK7, mean Nuclear DAB optical density (OD) of each core was automatically calculated. This resulted in continuous arbitrary variables to reflect protein expression of the tumor cells. For further analysis, mean value of the patients’ triplets was obtained. A representative subset of the cores was reviewed by two independent pathologists (CK and SP) with regard to a meaningful evaluation performed by the software. This ensured that expression above the calculated median corresponded conventional-morphologically to moderate to strong nuclear staining, and expression below the median to an absent or weak nuclear staining. Median of nuclear DAB OD (0.2497) from all samples was used to dichotomize the cohort into CDK7 high and low expressing group. On basis of this dichotomization, the statistical analysis concerning survival and correlation with other clinicopathological data was carried out.

### Statistical analyses

For the statistical analyses and data visualization, R software (version 4.0.2, R Foundation, Vienna, Austria; http://www.R-project.org) was used. T-tests were used to associate CDK7 expression with tumor entity and to examine differences between primary tumors with and without nodal metastases. To analyze a correlation of CDK7 expression with clinicopathological characteristics T-tests were applied. Chi-square tests were used to distinguish whether there were differences in clinicopathological features of the CDK7 low and CDK7 high expressing groups, which we considered in the survival analyses. Kaplan-Meier curves were used to illustrate overall survival (OS) and disease-free survival (DFS) in dependency of CDK7 expression and were statistically proved by log-rank tests. All tests were two-tailed and p-values of < 0.05 were considered statistically significant.

## Results

### CDK7 expression pattern in primary NSCLC

**Figure 1:**
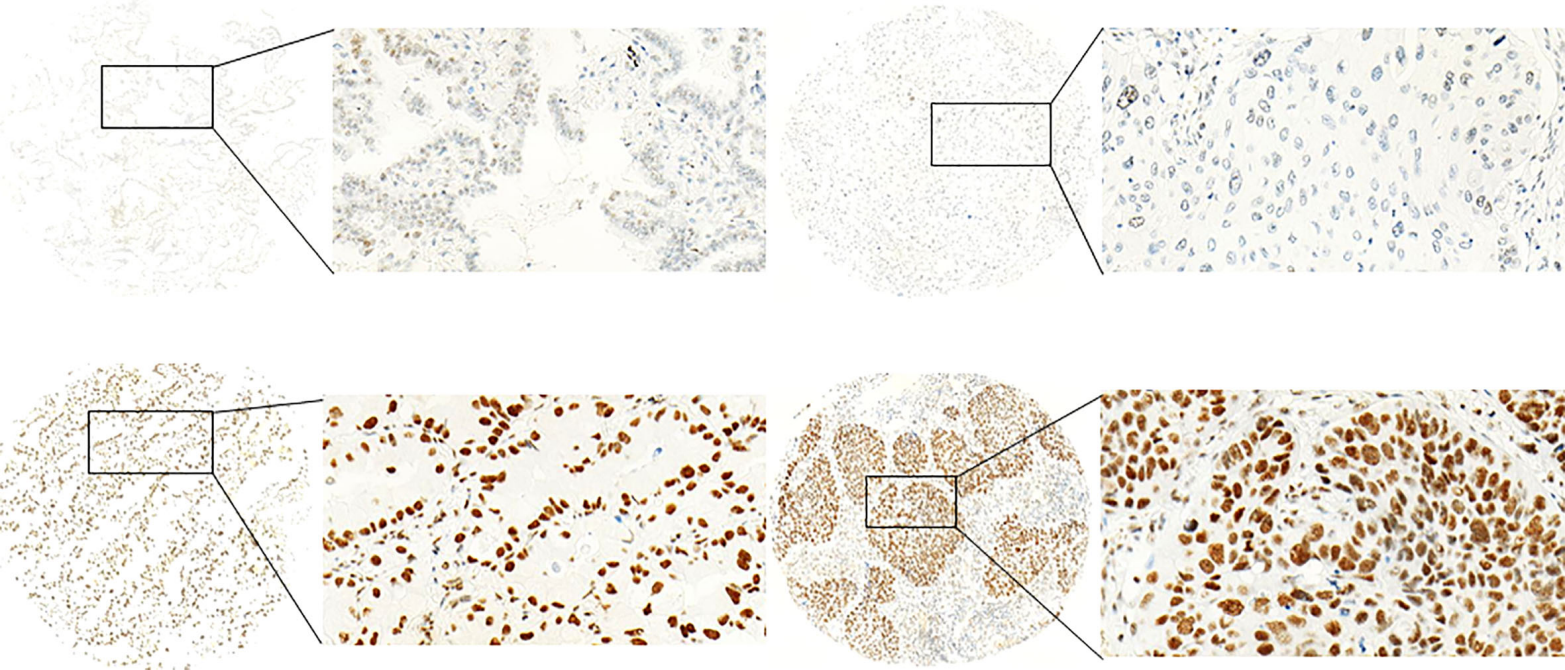
Exemplary pictures of CDK7 expression patterns in NSCLC. Left: adNSCLC with low (top) and high (below) expression (nuclear DAB OD 0.1547 and 0.5006, respectively). Right: sqNSCLC with low (top) and high (below) expression (nuclear DAB OD 0.1524 and 0.3969, respectively) of CDK7. Median nuclear DAB OD = 0.2497). The figures demonstrate specific nuclear staining that appears homogenous within the cores. (Objective magnification ×100 and 400, respectively).

CDK7 showed nuclear staining. Expression pattern for CDK7 between the cores originating from one tumor sample was homogenous meaning that the intratumoral difference in expression of the cells was neglectable. **Figure 1** provides pictures of immunohistochemical stainings. However, the overall expression between patients varied with a range of expression intensities from absent to strong immunoreactivity.

For further analyses, the cohort was divided in a CDK7 low and a CDK7 high expressing group by stratifying samples according to the median OD value of CDK7 (n= 179 each).

**Figure 2:**
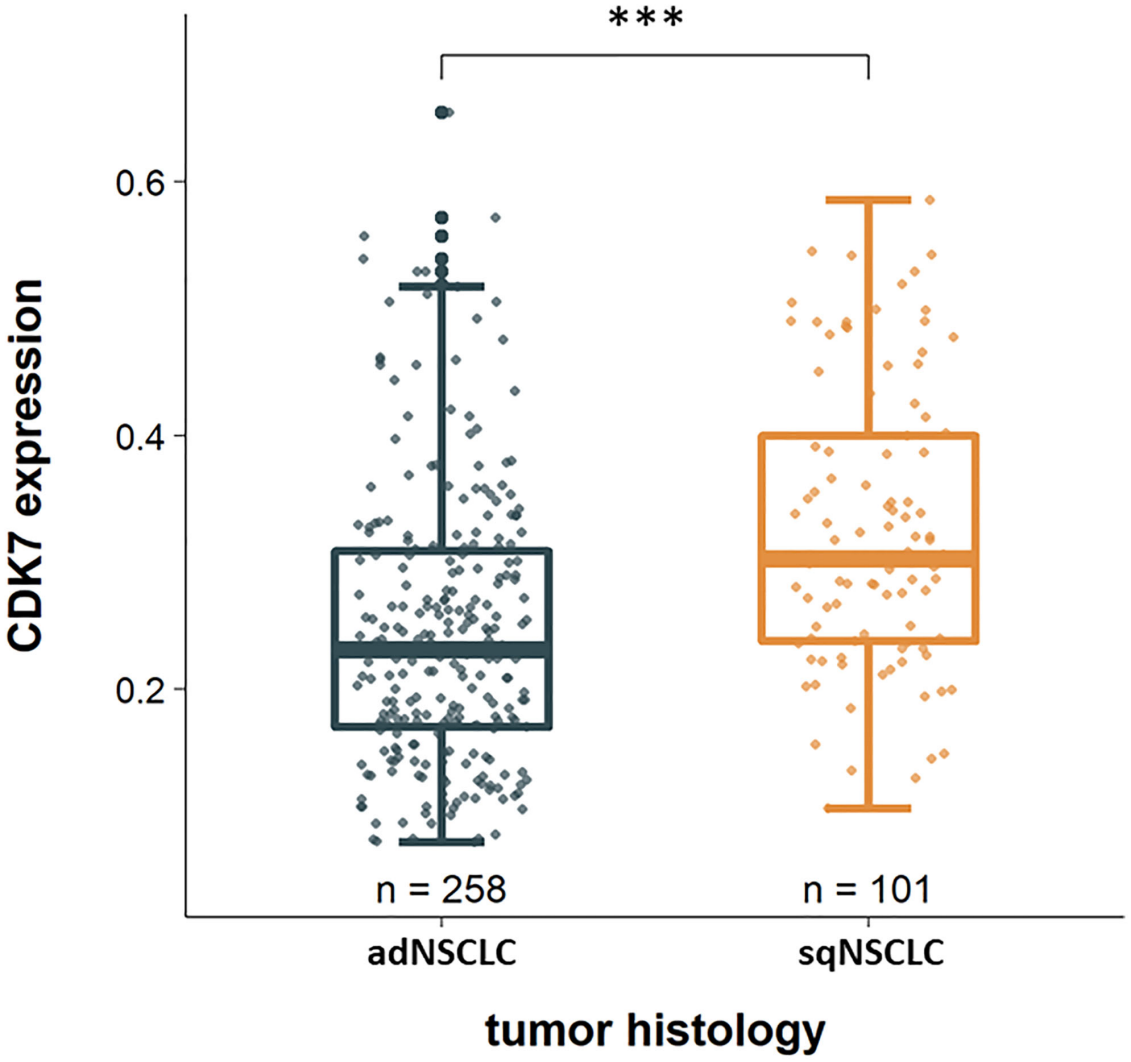
CDK7 expression in dependency of tumor histology. CDK7 is significantly higher expressed in sqNSCLC compared to adNSCLC (*** p <0.0001).

Considering the two groups of adNSCLC and sqNSCLC separately, one striking result was that CDK7 was significantly higher expressed in sqNSCLC than in adNSCLC (p <0.0001; **Figure 2**). Of the 101 sqNSCLC, 70 (69.3%) were CDK7 high and 31 (30.7%) were CDK7 low expressing tumors. Of the 258 adNSCLC instead, 109 (42.2%) were CDK7 high and 149 (57.8%) were CDK7 low expressing tumors.

### Correlation of CDK7 expression with clinicopathological characteristics

Concerning the whole cohort, there was no significant correlation of CDK7 expression concerning T-status, N-status, M-status, and UICC-status, grading, or time to recurrence. However, there was a trend for a higher UICC-stage (p 0.32) and worse grading (p 0.24) with increasing CDK7 expression (data not shown). Furthermore, CDK7 expression tended to be higher in primary carcinomas that later recurred compared to primary tumors that showed no relapse (p 0.34). One significant result was observed with regard to sex meaning that CDK7 was higher expressed in NSCLC of male patients (p=0.0274).

**Figure 3:**
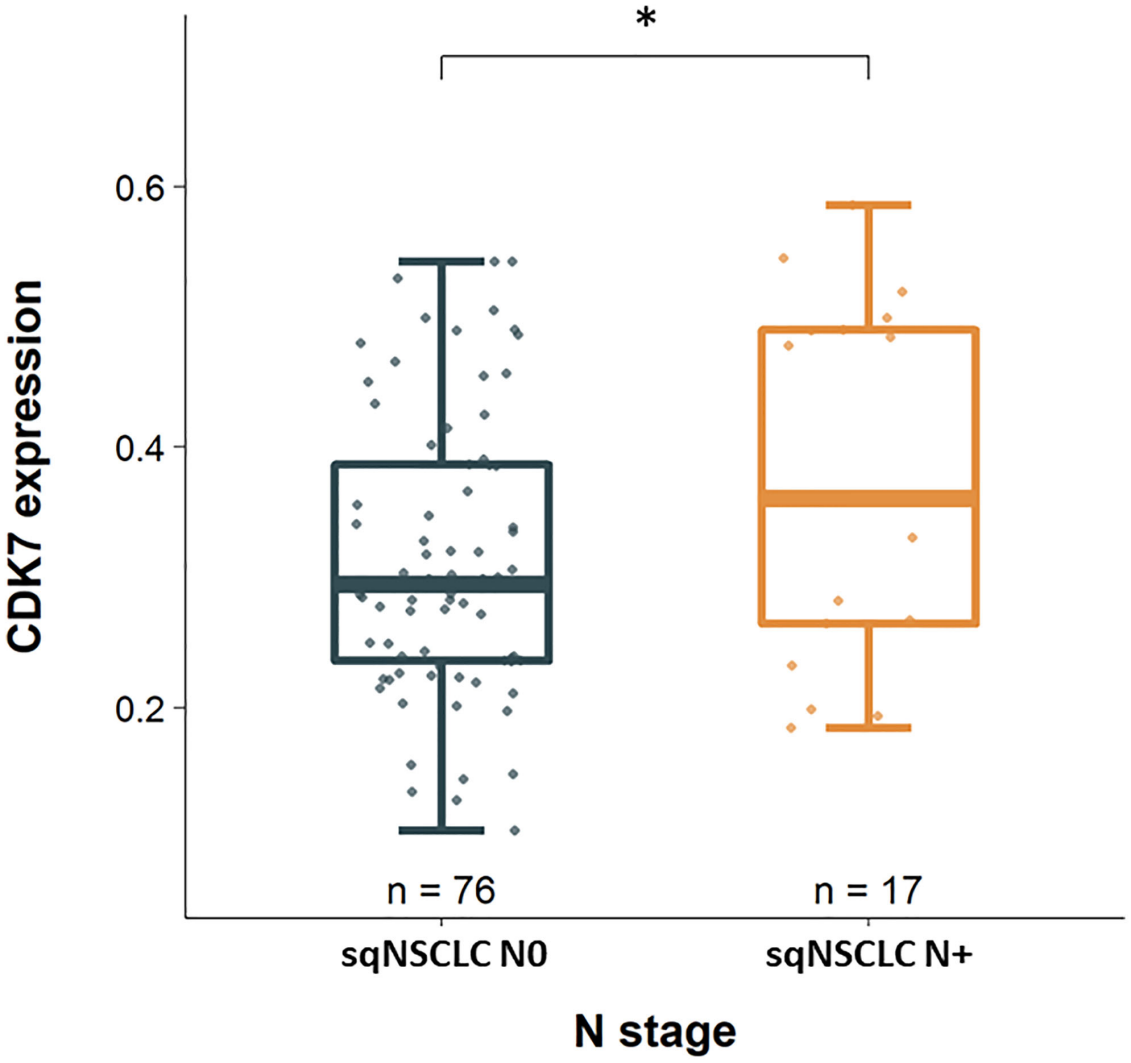
CDK7 expression between sqNSCLC with and without lymph node metastases. Compared to sqNSCLC with a pN0 stage, sqNSCLC with lymph node metastases show a significant higher CDK7 expression (* p 0.039).

Considering these parameters in the two groups of adNSCLC and sqNSCLC separately, we observed that CDK7 expression was significantly higher in sqNSCLC with lymph node metastases than in sqNSCLC with N0 stage (p 0.039; **Figure 3**). This could not be stated for the group of adNSCLC (p 0.086; not shown). There were no further significant differences concerning T-status, M-status, and UICC-status, grading or time to recurrence between the two entities with regard to CDK7 expression.

### Correlation of CDK7 expression with survival

We analyzed whether CDK7 expression could predict OS. Required follow-up data were available for the majority of the cohort of 346 cases (96.4%). By stratifying samples according to the median, two equal-size groups (high CDK7 expressing tumors above median (n=173) and low CDK7 expressing tumors below median (n=173)) were created (**Table 1**).

**Table 1:** Overview of clinico-pathological characteristics of CDK7 high and low expressing group.

	CDK7 low (n=173)	CDK7 high (n=173)	total (n=346)	p-value
**Sex**				0,159
female	82 (47.4%)	69 (39.9%)	151 (43.6%)	
male	91 (52.6%)	104 (60.1%)	195 (56.4%)	
**Age**				0,747
> median	84 (48.6%)	87 (50.3%)	171 (49.4%)	
< median	89 (51.4%)	86 (49.7%)	175 (50.6%)	
**T-stage**				0,517
missing	0	2	2	
T(1,2)	121 (69.9%)	125 (73.1%)	246 (71.5%)	
T(3,4)	52 (30.1%)	46 (26.9%)	98 (28.5%)	
**N-Stage**				0,29
missing	6	22	28	
N(0)	125 (74.9%)	105 (69.5%)	230 (72.3%)	
N(1,2)	42 (25.1%)	46 (30.5%)	88 (27.7%)	
**M-Stage**				0,814
missing	129	120	249	
M(0)	41 (93.2%)	50 (94.3%)	91 (93.8%)	
M(1)	3 (6.8%)	3 (5.7%)	6 (6.2%)	
**UICC-Stage**				0,507
missing	2	17	19	
Mean (SD)	1.719 (0.842)	1.782 (0.867)	1.749 (0.853)	
Range	1.000 - 4.000	1.000 - 4.000	1.000 - 4.000	
Median (Q1, Q3)	1.000 (1.000, 2.000)	2.000 (1.000, 2.250)	1.000 (1.000, 2.000)	
**Grading**				0,786
missing	1	2	3	
G(1,2)	87 (50.6%)	89 (52.0%)	176 (51.3%)	
G(3,4)	85 (49.4%)	82 (48.0%)	167 (48.7%)	

**Figure 4:**
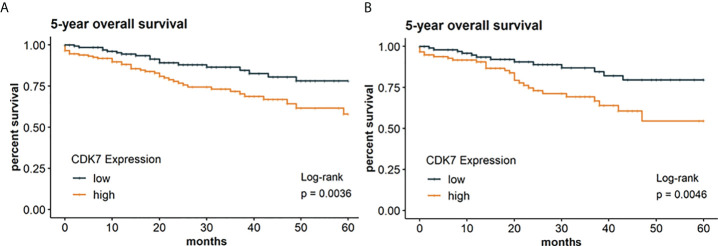
Kaplan Meier graphs with a p-value of Log-rank test of **(A)** 5-year overall survival of the whole cohort and **(B)** 5-year overall survival of the subcohort of adNSCLC. Median of CDK7 expression was used to stratify the cohort in two groups with expression above median considered as high expression and expression below median considered as low expression. Upregulation of CDK7 correlated significantly with a shorter OS for the whole cohort and group of adNSCLC (p=0.0036 and p=0.0046, respectively).

Concerning the whole cohort, Kaplan-Meier curve indicates a significantly worse OS for patients with CDK7 high expressing NSCLC than for patients with CDK7 low expressing NSCLC (log-rank test p 0.0036; **Figure 4A**). For OS, the 5-year survival rates were estimated at 58% for CDK7 high expression and 78% for low expression, respectively.

Due to expression of CDK7 was significantly different between adNSCLC and sqNSCLC, we assessed OS between both entities. We arrived at the same result for the subcohort of adNSCLC meaning that patients with CDK7 high expressing adNSCLC showed a significantly worse OS (p 0.0046; **Figure 4B**). 5-year survival rates were estimated at 55% for CDK7 high expressing adNSCLC and 80% for low CDK7 expressing adNSCLC.

For the subcohort of sqNSCLC there was no difference concerning OS in dependency of CDK7 expression (p 0.55; not shown).

**Figure 5:**
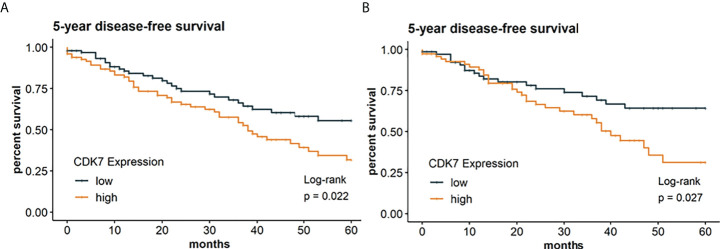
Kaplan Meier graphs with a p-value of Log-rank test of **(A)** 5-year disease-free survival of the whole cohort and **(B)** 5-year disease-free survival of the subcohort of adNSCLC. Median of CDK7 expression was used to stratify the cohort in two groups with expression above median considered as high expression and expression below median considered as low expression. Upregulation of CDK7 correlated significantly with a shorter DFS for the whole cohort and group of adNSCLC (p=0.0022 and p=0.0027, respectively).

In the following, we investigated if CDK7 expression has also an implication regarding DFS thereby taking the whole cohort as well as the subcohorts separately into consideration. Survival analysis was restricted to 202 patients with required follow-up data. According to the median, Kaplan-Meier curve indicates a significantly worse DFS for patients with CDK7 high expression in NSCLC than for patients with CDK7 low expression in NSCLC (log-rank test p 0.022; **Figure 5A**). 5-year disease-free survival rates were 32% and 56% for high and low CDK7 expression, respectively.

The same applies to the group of adNSCLC (p 0.027; **Figure 5B**). Here, 5-year survival rates were estimated at 31% for CDK7 high expressing adNSCLC and 64% for low CDK7 expressing adNSCLC.

As for OS, we also found no difference of DFS in dependency of CDK7 expression for the subcohort of sqNSCLC (p 0.15; not shown).

To assess if the prognostic value of CDK7 expression was independent of other prognostic factors for 5-year OS and 5-year DFS, at first univariable followed by multivariable cox regression was performed. It was found that high CDK7 expression was not an independent prognostic factor neither for OS nor for DFS (OS: HR=4.92 (95% CI 0.92-26.41), p=0.063; DFS: HR= 0.94 (95% CI 0.47-1.89), p=0.858).

## Discussion

Despite promising advances in the therapy of NSCLC, the identification of new prognostic and therapeutically targetable biomarkers is needed. Molecular targeted therapy has improved the survival of adNSCLC patients, while less advances have been made in the treatment of sqNSCLC (18). Recently, transcription-associated CDKs, to which CDK7 also belongs, have emerged as an exciting area of great promise in oncology. Various studies demonstrate that cancers depend on oncogenic transcription factors and their downstream networks can be therapeutically targeted by CDK7 inhibitors of which THZ1 is the best known (2). The role of CDK7 in NSCLC has not yet been investigated as thoroughly as in other malignancies, and concerning lung cancer, there are already more insights for SCLC than for NSCLC. However, for NSCLC, there are few *in vitro* studies using cell lines from pulmonary adenocarcinoma and squamous cell carcinoma demonstrating that THZ1 effectively inhibits cell proliferation and migration, causes cell-cycle arrest, induces apoptosis, and suppresses glycolysis (1, 16–18). Wang et al. (1) have conducted extensive *in vitro*- and *in vivo*- studies and could demonstrate that CDK7 silencing and inhibition with THZ1 elicited apoptosis and suppressed tumor growth of NSCLC. Moreover, THZ1 boosted antitumor immunity by recruiting infiltrating CD8+ T cells and synergized with anti-PD-1 therapy. The authors conclude that the combined CDK7 inhibition and anti-PD-1 therapy could be an effective treatment of NSCLC.

However, no direct link between protein expression of CDK7 and sensitivity towards CDK7 inhibition is mentioned in the studies. There are only few studies dealing with protein expression of CDK7 on lung cancer. To the best of our knowledge, this should be the first study to investigate protein expression of CDK7 in a large cohort of NSCLC containing both adNSCLC and sqNSCLC, which represent the two major subtypes of NSCLC (approx. 60 and 30%, respectively) (18, 22).

### CDK7 expression pattern

Concerning evaluation of expression pattern, we set a high and low expressing group according to the median nuclear DAB OD value of CDK7 resulting in two groups of equal size (n=179 each). Automatically calculated values reflecting protein expression of the tumor cells were reviewed with regard to a meaningful evaluation by two pathologists. Expression values above the calculated median corresponded conventional-morphologically to moderate to strong nuclear staining, and expression below the median to an absent or weak nuclear staining.

Our observed expression pattern fits into data in literature. For example, in a study by Wang et al., the group sizes of CDK7 low and high expressing adNSCLC in two independent cohorts were also approximately equal (cohort 1: CDK7 low n=52 (56.5%), CDK7 high n=40 (43.5%); cohort 2: CDK7 low n=118 (53.2%), CDK7 high n=104 (46.8%)). In this regard, assessment of immunohistochemical staining must be taken into account. In above-mentioned study, evaluation of CDK7 staining pattern is comparable to ours. CDK7 staining was semi-quantified scored as 0 to 3+ using signal intensity in the tumor cell nuclei (no staining = 0, weak = 1, moderate = 2, strong = 3). CDK7 score ≥ 2 was used as cut-off for dividing the samples into a high and low expression group. In another study dealing with CDK7 expression on adNSCLC corresponding group sizes are not explicitly stated (22).

There are seemingly so far no published studies concerning protein expression of CDK7 in sqNSCLC. However, there are studies that have investigated squamous cell carcinomas (SCC) of other sites. Jiang et al. examined protein expression of CDK7 in oral squamous cell carcinomas (OSCCs) (n=113). Immunoreactivity was evaluated using the immunoreactive score reaching from 0 to 12 (intensity score × proportion score defining intensity score as 0 = negative, 1 = weak, 2 =moderate, 3= strong and defining proportion score as 0 = negative, 1= <10%, 2 = 11-50%, 3 = 51-80%, 4 = >80% positive cells). The distribution of the samples in a CDK7 low and high expressing group was 54.8% (n=62) and 45.2% (n=51), respectively and was thus also even. Zhang et al. who examined protein expression of CDK7 in 98 esophageal squamous cell carcinomas (ESCC) used the same score and with that found a higher proportion of CDK7 high expressing tumors (81.6%, n=80).

We found that CDK7 was significantly higher expressed on sqNSCLC than on adNSCLC (**Figure 2**). Since there are no other studies that have comparatively investigated the protein expression of CDK7 on both entities, our results are not comparable one to one.

### Prognostic significance

Concerning survival data, we found a high CDK7 protein expression to be associated with a poor OS and DFS for the whole cohort and the group of adNSCLC (**Figures 4** and **5**). This finding is in line with that of Wang et al. In this already above-mentioned study, two independent cohorts of pure adNSCLC (n = 92 and 222, respectively) were investigated (1). The authors stated a significantly poorer OS (p 0.036 and 0.003, respectively) in adNSCLC with high CDK7 protein expression. Bian et al. (22) examined 100 samples of adNSCLC *via* immunohistochemistry and also found a significantly poorer OS for patients with high expression of CDK7 (p < 0.0001).

In agreement with these findings, studies investigating protein expression of CDK7 in squamous cell carcinomas stated a relationship of increased CDK7 expression with poor prognosis. Since no studies concerning protein expression of CDK7 in sqNSCLC are published so far, literature regarding CDK7 expression on squamous cell carcinomas (SCC) of other sites is discussed. Jiang et al. found an elevated CDK7 expression on OSCCs to be significantly associated with a reduced OS as well as DFS (p 0.022 and 0.010, respectively) (7). Zhang et al. (9) stated that patients with CDK7 high expressing ESCC had a significantly shorter OS (p 0.01) and identified CDK7 as an independent prognostic indicator of OS. Jagomast et al. (8) also noted that CDK7 overexpression resulted in significantly worse 5-year OS as well as DFS rates for HNSCC patients (p 0.037 and 0.016, respectively). Since aetiological factors for sqNSCLC include first of all smoking which also applies to OSCC, ESCC, and HNSCC a comparison of CDK7 expression between these carcinoma entities is justified.

Just mentioned results are in contrast to our finding that there was no significant association of CDK7 expression with regard to OS and DFS in the subcohort of sqNSCLC. Here, the relatively smaller number of cases compared to the number of adNSCLC in our cohort (n= 101 vs. 258) should be taken into consideration. Still, the reasons are not entirely comprehensible especially since the group size of sqNSCLC (n=101) is comparable to SCC cohorts in literature and since etiologic factors overlap (7, 9). Possible reasons could be different definitions for high vs. low expressing tumors (cf (7, 9).) and a smaller cohort size compared to the study of Jagomast et al. (n=419) who used the same definition for CDK7 low vs. CDK7 high (8). Whether CDK7 is indeed no prognostic for sqNSCLC should be investigated on independent and larger sqNSCLC cohorts.

Interestingly, we found an overall higher expression of CDK7 for sqNSCLC than for adNSCLC (**Figure 2**), for which we could not demonstrate an association with survival data. In addition, for sqNSCLC we found a higher expression of CDK7 in primary tumors that were nodally metastatic than in non-metastatic sqNSCLC (**Figure 3**). The results were significant (p 0.039) even though the proportion of sqNSCLC with metastases was smaller than the proportion of sqNSCLC without metastases (n=17 (18.3%) vs n=76 (81.7%), **Figure 3**). Since metastasis generally results in poorer survival, the data appear contradictory. Here, it should be taken into account that the group of sqNSCLC (n=101) might be too small to provide an association with survival data. However, this finding may suggest that immunohistochemically detected overexpression of CDK7 on sqNSCLC is indicative of lymph node metastasizing and thus greater aggressiveness. These findings need further analysis on larger cohorts of sqNSCLC. Studies investigating CDK7 expression on SCC did not discover a significant correlation with N-Stage (7–9).

There was no significant association of CDK7 expression in dependency of lymph node metastasizing in the subcohort of adNSCLC (p 0.086) although the proportion of metastatic adNSCLC (n=77; 32.8%) among adNSCLC was higher than the proportion of metastatic sqNSCLC among sqNSCLC. Studies that have investigated CDK7 expression on adNSCLC do not address correlation with metastasis, so no comparison with our data can be made here.

### Correlation of CDK7 Expression with other clinicopathological variables

The only significant correlation was found in relation to sex meaning that CDK7 was higher expressed in NSCLC from male patients (p=0.0274). This observation should however be primarily a coincidence without having a causal relationship. Our observation that CDK7 expression does actually not associate with clinicopathological data other than survival broadly fits with those from cited literature. However, for example, Jiang et al. found an elevated CDK7 expression on OSCCs to be significantly associated with higher T-stage (p 0.009) (7) and Zhang et al. stated that overexpression of CDK7 was significantly associated with worse tumor grade (p<0.01). Studies on protein expression of CDK7 on adNSCLC do not explicitly address its correlation with clinic-pathological parameters on which we focused (1, 22).

In summary, to our knowledge, we are seemingly the first to examine CDK7 protein expression on a cohort of NSCLC containing both adNSCLC and sqNSCLC. These data are of interest due to promising *in vitro* studies on CDK7 and its inhibition in NSCLC exist already. We found CDK7 be to significantly higher expressed on sqNSCLC than on adNSCLC. CDK7 expression was significantly higher in sqNSCLC with lymph node metastases than in sqNSCLC with N0 stage, indicating a greater aggressiveness. We found a significantly worse OS and DFS for CDK7 high expressing tumors in the whole NSCLC cohort and subcohort of adNSCLC.

Our findings suggest that the CDK7 protein expression status could offer valuable information about prognosis of NSCLC patients and thereby it could serve as indicator for a meaningful follow-up management. Therefore, its involvement in routine diagnostic workup of NSCLC samples could be meaningful in the future. Additionally, due to its clear nuclear staining, expression pattern would be effortless to evaluate.

Our study has the major limitation that our results are not validated with an independent cohort. Since the results of a single biomarker study have limited value, validation from other researchers on independent cohorts is required.

Given promising previous findings of *in vitro* studies with CDK7 inhibitors on NSCLC cells together with our findings and assuming that protein expression of CDK7 in the large cohort of NSCLC might indicate sensitivity to CDK7 inhibition, one could suggest that CDK7 might serve as a novel prognostic biomarker and additionally as therapeutic target.

Finally, prospective studies are necessary to validate our findings on independent cohorts, to investigate a correlation between CDK7 protein expression and sensitivity towards CDK7 inhibition, and to unravel molecular mechanisms by which CDK7 contributes to poor prognosis in NSCLC.

## Data availability statement

The raw data supporting the conclusions of this article will be made available by the authors, without undue reservation.

## Ethics statement

The study was conducted in accordance with the Declaration of Helsinki, and the protocol was approved by the local ethics council at the University of Lübeck (file number 16-277, 16-278). For the present study, the retrospective part (16-277) of the ethics application applies. It stipulates that statement on consent of the patients for research purposes is required for samples from 2018 onwards. The samples examined in this study originated from earlier years (2005-2017).

## Author contributions

SP and CK planned the research project. ED performed the immunohistochemical stainings. TJ performed the statistical analysis. CH, F-OP, SB, and SS provided patients’ follow-up data. CK, TJ, CH, F-OP, SB, SS, JK, and SP wrote and/or revised the manuscript. All authors have read and agreed to the published version of the manuscript.

## Funding

Supported with funds from the Section of Medicine at the University of Luebeck J18-2020.

## Conflict of interest

SP is a consultant of Ventana, Roche, Novartis, Astellar, Astrazeneca, Bristol-Myers Squibb, Merck Serono and MSD. JK is a consultant of Roche, AMGEN and BMS.

The remaining authors declare that the research was conducted in the absence of any commercial or financial relationships that could be construed as a potential conflict of interest. The above-mentioned companies had no influence on the study design, acquisition of data, or writing of the manuscript.

## Publisher’s note

All claims expressed in this article are solely those of the authors and do not necessarily represent those of their affiliated organizations, or those of the publisher, the editors and the reviewers. Any product that may be evaluated in this article, or claim that may be made by its manufacturer, is not guaranteed or endorsed by the publisher.

## Supplementary material

Supplementary Figure 1 Exemplary TMA core of a CDK7 stained sqNSCLC segmented automatically by QuPath (tumor cells are annotated with yellow/orange depending on chromogenic intensity, immune cells are annotated with purple/blue, stroma with green, pigment with black). Tumor cells were defined as regions of interest.

## Abbreviations

adNSCLC, pulmonary lung adenocarcinoma; CDKIs, CDK inhibitors; CDK7, Cyclin-depending Kinase 7; DFS, disease-free survival; ESCC, esophageal squamous cell carcinomas; FFPE, Formalin-fixed paraffin-embedded; HNSCC, head and neck squamous cell carcinomas; IHC, Immunohistochemistry; OD, optical density; OS, overall survival; OSCCs, oral squamous cell carcinomas; SCC, squamous cell carcinoma; sqNSCLC, pulmonary squamous cell carcinoma; TMA, tissue microarrays.
