# Supplementary figures and images for: CDK7 is a prognostic biomarker for non-small cell lung cancer
Source: Front Oncol. 2022 Sep 23;12:927140. doi: 10.3389/fonc.2022.927140 (PMC9540232; doi:10.3389/fonc.2022.927140)

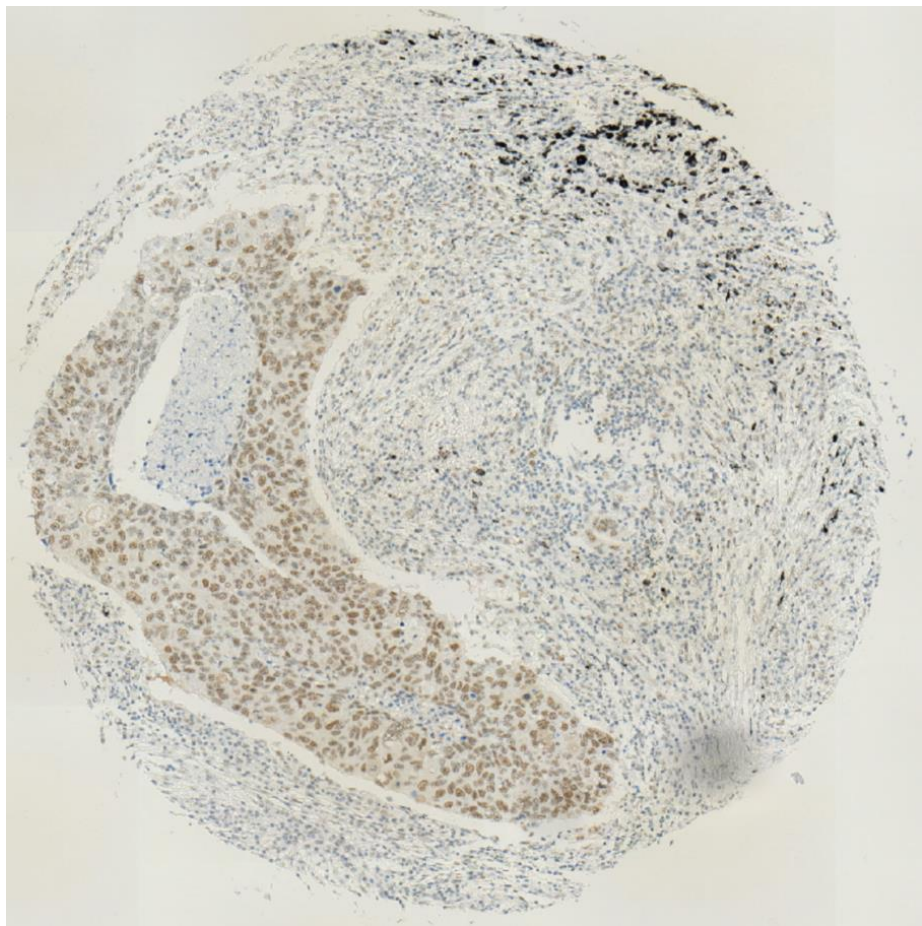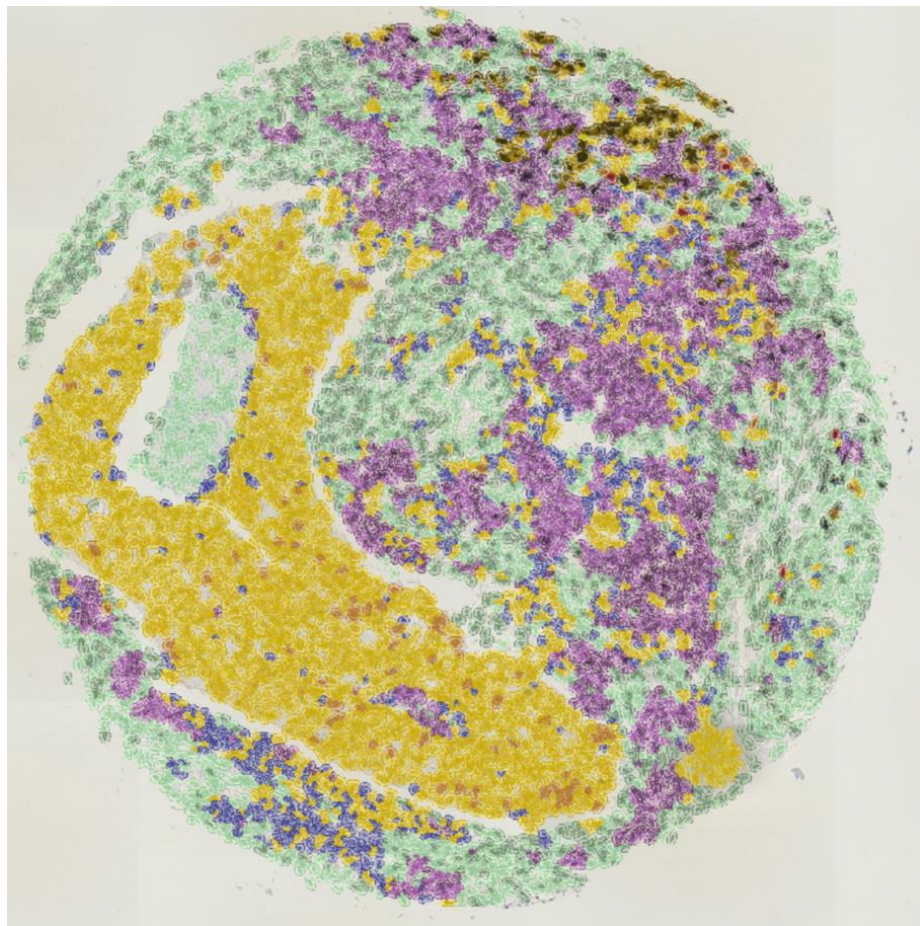

Supplement: Supplementary Figure 1 — Exemplary TMA core of a CDK7 stained sqNSCLC segmented automatically by QuPath (tumor cells are annotated with yellow/orange depending on chromogenic intensity, immune cells are annotated with purple/blue, stroma with green, pigment with black). Tumor cells were defined as regions of interest. [file Image_1.pdf]
